# Cisplatin Relocalizes RNA Binding Protein HuR and Enhances the Oncolytic Activity of E4orf6 Deleted Adenovirus

**DOI:** 10.3390/cancers12040809

**Published:** 2020-03-27

**Authors:** Umma Habiba, Elora Hossain, Aya Yanagawa-Matsuda, Abu Faem Mohammad Almas Chowdhury, Masumi Tsuda, Asad-uz- Zaman, Shinya Tanaka, Fumihiro Higashino

**Affiliations:** 1 Department of Cancer Pathology, Faculty of Medicine, Hokkaido University, Sapporo 060-8638, Japan; 2 Department of Oral Pathology and Periodontology, Sapporo Dental College and Hospital, Dhaka 1230, Bangladesh; 3 Department of Molecular Oncology, Hokkaido University Faculty of Dental Medicine and Graduate School of Biomedical Science and Engineering, Sapporo 060-8586, Japan; 4 Department of Vascular Biology and Molecular Pathology, Hokkaido University Faculty of Dental Medicine and Graduate School of Dental Medicine, Sapporo 060-8586, Japan; 5 Department of Restorative Dentistry, Hokkaido University Faculty of Dental Medicine, Sapporo 060-8586, Japan; 6 Institute for Chemical Reaction Design and Discovery (WPI-ICReDD), Hokkaido University Sapporo, Sapporo 001-0021, Japan; 7 Global Institution for Collaborative Research and Education (GI-CoRE), Hokkaido University Sapporo, Sapporo 060-0808, Japan

**Keywords:** ARE, AU-rich element, HuR, human antigen R, 3′-UTR, 3′-untranslated region, adenovirus, Cis-diamminedichloroplatinum (CDDP)

## Abstract

The combination of adenoviruses and chemotherapy agents is a novel approach for human cancer therapeutics. A meticulous analysis between adenovirus and chemotherapeutic agents can help to design an effective anticancer therapy. Human antigen R (HuR) is an RNA binding protein that binds to the AU-rich element (ARE) of specific mRNA and is involved in the export and stabilization of ARE-mRNA. Our recent report unveiled that the E4orf6 gene deleted oncolytic adenovirus (dl355) replicated for certain types of cancers where ARE-mRNA is stabilized. This study aimed to investigate whether a combined treatment of dl355 and Cis-diamminedichloroplatinum (CDDP) can have a synergistic cell-killing effect on cancer cells. We confirmed the effect of CDDP in nucleocytoplasmic HuR shuttling. In vitro and in vivo experiments showed the enhancement of cancer cell death by apoptosis induction and a significant reduction in tumor growth following combination treatment. These results suggested that combination therapy exerted a synergistic antitumor activity by upregulation of CDDP induced cytoplasmic HuR, which led to ARE mRNA stabilization and increased virus proliferation. Besides, the enhanced cell-killing effect was due to the activation of the intrinsic apoptotic pathway. Therefore, the combined treatment of CDDP and dl355 could represent a rational approach for cancer therapy.

## 1. Introduction

Currently, oncolytic adenoviruses are being developed as novel antitumor therapeutics. To enhance the therapeutic potential, adenoviruses are being administered alongside standard chemotherapy. For the replication of adenovirus, several early gene products, such as E1A, E1B, and E4, are necessary to change the host tumor microenvironment [1]. The E4 region encoding proteins such as E4orf1, E4orf3, and E4or6, are known to have oncogenic activities that are required for DNA replication, late gene expression, and host cell shutoff [2,3,4,5,6,7]. E4orf1 of adenovirus type 9 (subgroup D) is involved in mammary tumorigenesis [4,8]. E4orf3 and E4orf6 proteins of adenovirus type 5 (subgroup C) have the potentials to transform cells in cooperation with E1A and E1B and intensify the growth of tumors transplanted in nude mice [5,6,7]. In our previous report, we confirmed that in cells transfected with adenovirus type 5, E4orf6 associated with cellular pp32 protein and AU-rich element (ARE)-containing mRNAs were exported to the cytoplasm in a chromosome region maintenance 1 (CRM1)-independent manner [9]. Furthermore, the exported ARE-mRNAs were stabilized and acquired the potential to transform cells [10,11].

AREs usually exist in the 3’-untranslated region (3′-UTR) of specific mRNAs encoding genes required for cell growth and proliferation and enhance the rapid decay of mRNA [12,13]. Several RNA binding proteins control the fate of ARE mRNA. For example, HuR binds to AREs to protect ARE-mRNA from rapid degradation, whereas tristetraprolin (TTP), AUF1, accelerated the degradation of ARE-mRNA [12,14,15]. HuR generally localized in the nucleus but can shuttle between the nucleus and cytoplasm. HuR-induced stabilization of ARE-mRNA depends on HuR localization in the cytoplasm [15,16]. In normal cells, cytoplasmic translocation of HuR is transient; on the other hand, HuR constitutively accumulates in the cytoplasm of cancer cells. Cytoplasmic HuR may contribute to the malignant phenotype of cancer and thought to be involved in malignant transformation [16,17]. 

CDDP is one of the commonly used anticancer drugs for the treatment of human malignant tumors. However, drug resistance and severe side effects are the major clinical hurdles that remain associated with CDDP- based treatment [18,19]. Few studies reported increased patient survival, more cancer cell death, less toxicity when applied with adenovirus combination with CDDP. However, the detailed mechanism and appropriate combination are yet to develop [20,21]. 

dl355 is an E4orf6-deleted mutant adenovirus which has a 14bp-deletion in the E4orf6 gene. In a recent study, we characterized the cell death efficacy of dl355 and made a comparison between virus production and cell lysis activity between dl355 and dl1520/ ONYX-015. The result showed that dl355 selectively target cancer cells and has a more substantial oncolytic effect than dl1520 (22). The study used the normal cells (BJ) and wild type virus (WT300) as a control.

We previously demonstrated the role of HuR in ARE mRNA stabilization that lead to dl355 propagation [22]. Therefore, the objective of this study was to evaluate whether combination therapy with dl355 and CDDP would show enhanced anti-tumor effect against different cancer. In this study, we confirmed the effect of CDDP in cytoplasmic HuR translocation. In combination therapy, we observed increased cell killing and increased virus replication both in vitro and in vivo. This study demonstrates the enhanced antitumor efficacy of combination therapy for the first time, where virus replication is dependent on ARE mRNA stabilization.

## 2. Results

### 2.1. Characterization of E4orf6 Gene Deleted Mutant Adenovirus and Cancer Cell Death Efficacy

**Figure 1:**
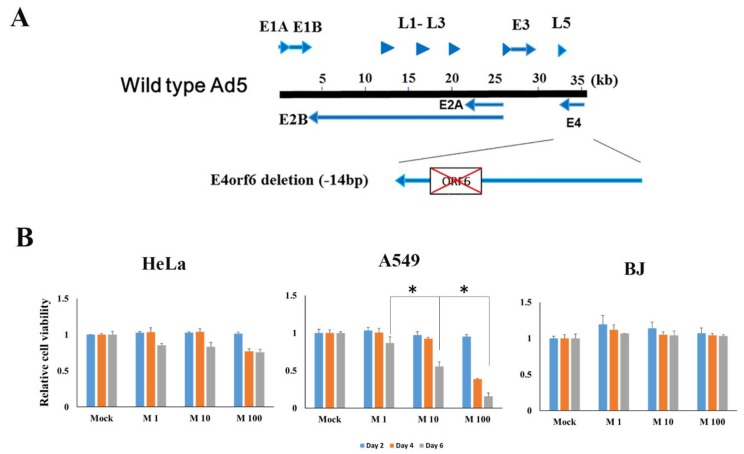
Schematic representation of dl355 and its cancer cell death efficiency. (**A**). Schema of dl355 shows a 14 base-pair (bp) deletion in the open reading frame 6 gene of the wild type adenovirus type 5 (Ad5) E4 region. Arrows indicate early genes (E1-4) and arrowheads indicate late (L1-5) genes. (**B**). Cancer (HeLa and A549) and normal (BJ) cells were infected with dl355 at indicated MOIs (1, 10, 100; vp/cell). The cell viability was measured by the XTT assay. The result shown is from a single experiment representative of two similar experiments. * indicates *p* < 0.05.

dl355 is an E4orf6 gene deleted mutant adenovirus, which has a 14bp-deletion in the E4orf6 gene (Figure 1A). The functions of different genes in the E4 region was evaluated and found that dl355 showed impaired virus DNA replication, accumulation of late viral mRNAs, and shutoff of host cell mRNAs compared to wild type adenovirus type 5 [2]. Our recent study reported that HuR mediated ARE-mRNA stabilization enhanced dl355 replication.

To evaluate the cell lysis activity of dl355, cancer (HeLa and A549) and normal (BJ) cells were infected with dl355 (MOI 1,10, 100; vp/cell), and the cell viability was observed at different time intervals (days 2, 4, and 6). As shown in Figure 1B, dl355 infection resulted in a dose and time-dependent reduction of cell viability in cancer cells, although cell lysis activity was different in individual cancer. A549 cells showed a significant decrease in cell viability over time, whereas BJ cells survived even after six days. Taken together, these results demonstrate the cancer-selective cytolytic activity of dl355. As A549 cells showed a cytopathic effect at the concentration of MOI 10 after day 2 of post-infection, this concentration was selected for further experiments.

### 2.2. Modulatory Effect of Nucleocytoplasmic HuR Translocation by CDDP Treatment

**Figure 2:**
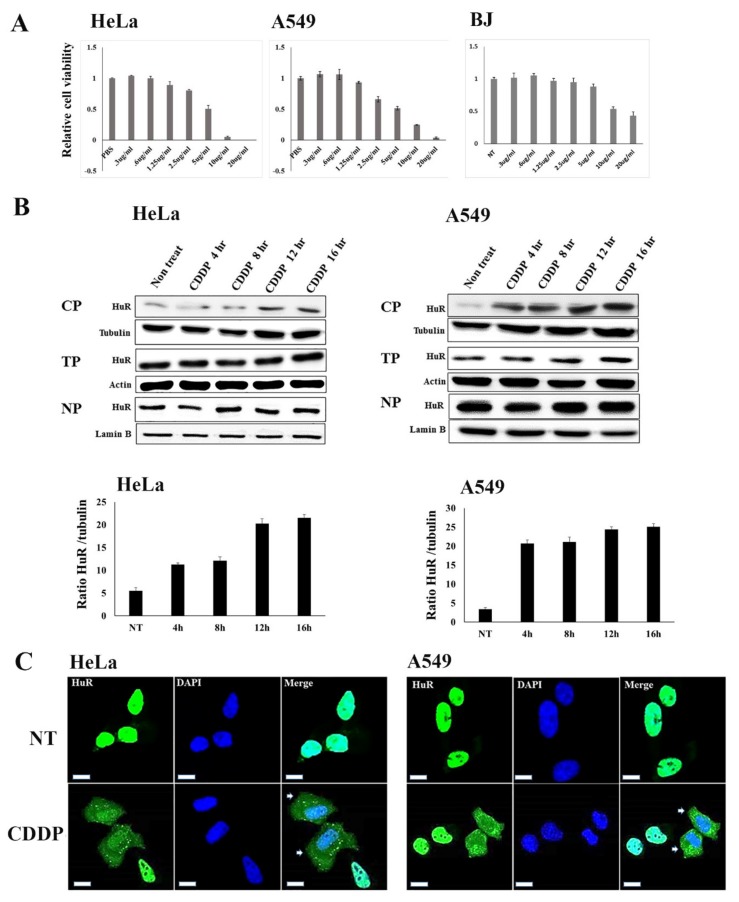
The effect of CDDP in nucleo-cytoplasmic HuR translocation. (**A**). Cancer (HeLa and A549) and normal (BJ) cells were treated with CDDP for 48 h, and the XTT assay measured cell viability. (**B**). Cancer cells (HeLa and A549) were treated with CDDP (1.25 µg/mL) for 4 to 16 h. The level of HuR was monitored in the total (TP), cytoplasmic (CP), and nuclear (NP) protein. The bar chart shows the quantification of the cytoplasmic HuR level compared to tubulin in each condition (lower panel). (**C**). Cancer cells (HeLa and A549) were treated with CDDP, as mentioned above. For the visualization of nucleocytoplasmic translocation, cells were stained with anti-HuR primary and Alexa-Fluoro 488 secondary antibody. Arrow marks indicate cytoplasmic HuR expression, and granular structure meant stress granule (SG). DAPI (40, 60-DAPI) was used to visualize cell nuclei. Bars, 20 µm. The result shown is from a single experiment representative of two similar experiments.

The cell viability of cancer (HeLa and A549) and normal (BJ) cells was assessed by the XTT assay. Cells were treated with CDDP at different concentrations ranging from 0.3 to 20 µg/mL for 48 h. CDDP treatment resulted in a dose-dependent reduction of cell viability (Figure 2A). As the cells started to die at the concentration of 1.25 µg/mL, we selected this dose for further experiments.

The effect of CDDP on nucleocytoplasmic HuR translocation was assessed after treating the Hela and A549 cells for 4 to 16 h. HuR expression began to increase in 4 hours (A549) and 8 h (HeLa) after the treatment with CDDP. However, the total and nuclear content of HuR remained unchanged in both cell types (Figure 2B). 

Later we performed the confocal microscopy to visualize nucleocytoplasmic translocation of HuR. Cytoplasmic extension of HuR staining was observed in CDDP treated, whereas the staining mainly localized in the nuclei of untreated cells (Figure 2C). In addition, we found the granular stress granule (SG) in CDDP treated cancer cells, which shows enhanced cytoplasmic HuR protein. From these observations, we suggested that CDDP has an effect on the cytoplasmic HuR translocation, and the upregulation of cytoplasmic HuR might be linked to SG formation.

### 2.3. Effect of Combination Therapy on Cell Viability

**Figure 3:**
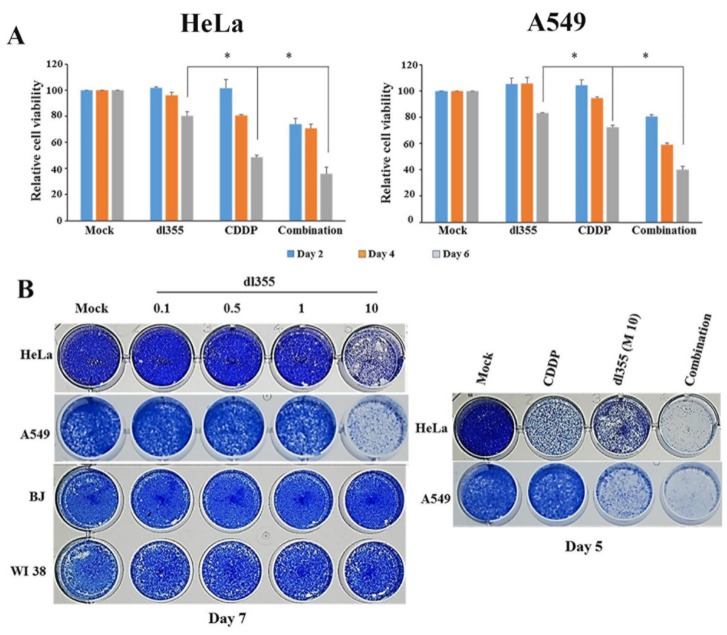
Cell viability assessment after dl355 infected and combination therapy. Cells were infected with dl355 at indicated MOIs (0.1, 0.5, 1, 10; vp/cell) or treated with CDDP (1.25 µg/mL) or combination (CDDP 1.25 µg/mL plus dl355 MOI 10; vp/cell) therapy. For combination therapy, cells were treated with CDDP for 4 h before infection with the virus. (**A**) At 2, 4, and 6 days of post-infection, cell viability was calculated by the XTT assay. (**B**) At 5 and 7 days of post-treatment, coomassie brilliant blue staining was used to evaluate the cytopathic effect. The result shown is from a single experiment representative of two similar experiments. * indicates *p* < 0.05.

The role of HuR in ARE-mRNA stabilization and enhancement of dl355 replication was confirmed in a previous study [22]. For the combination treatment, we selected 1.25 µg/mL and MOI 10; vp/cells for the CDDP and dl355, respectively. As HuR began to translocate to the cytoplasm in four (A549) and 8 h (HeLa) after the treatment with CDDP (Figure 2B), we treated the cells with CDDP for 4 h before infection with the virus for in vitro combination therapy. At 2, 4, and 6 days of treatment, cell viability was calculated by the XTT assay. Combination treatments showed a significantly higher cytotoxic effect compared with cells that were treated with CDDP or dl355 alone. As shown in Figure 3A, in the HeLa cell line, dl355 and CDDP resulted in cell killing of 20% and 55%, respectively, whereas combination therapy resulted in 64% cell killing. On the other hand, in the A549 cell line, dl355 and CDDP resulted in cell killing of 17% and 28%, respectively, whereas in combination therapy resulted in 60% cell killing. 

To evaluate the potential interactions between the drug and the virus, the Chou-Talalay combination indices (CI) of the CDDP in combination with dl355 were analyzed. CI of < 0.9 indicates synergy, CI between 0.9 and 1.1 is addictive, and CI of >1.1 indicates antagonism. For the combination of dl355 and CDDP, Chou-Talalay CIs ranged from 0.21 to 0.47 for HeLa and 0.26 to 0.85 for A549, demonstrating synergistic cytotoxicity (Figure S1). 

Cytopathic effects (CPE) assay was performed to assess the cell lysis activity of dl355 and to evaluate whether combination therapy is similarly effective in other cancer types besides HeLa and A549, we included five different cancer (U2OS, HSC3, Ca922, H1299, and C33A) and two normal cell types (BJ and WI38). Cells were infected with dl355 at indicated MOIs (0.1, 0.5, 1, 10; vp/cell) and incubated for 7 days. For combination therapy, a similar protocol was used, as mentioned above, and incubated for five days. At 5 and 7 days of post-treatment, coomassie brilliant blue staining was used to evaluate the cytopathic effect. CPE assay revealed that cell lysis activity of dl355 was different in individual cancer, and combination therapy could kill cancer cells within a quicker duration than each treatment alone (Figure 3B and Figure S2). Together, these in vitro data demonstrate that the combination therapy results in enhanced cell killing for cancer cells where ARE-mRNA is stabilized. 

### 2.4. Combination Therapy Augments Apoptosis in Cancer Cells

**Figure 4:**
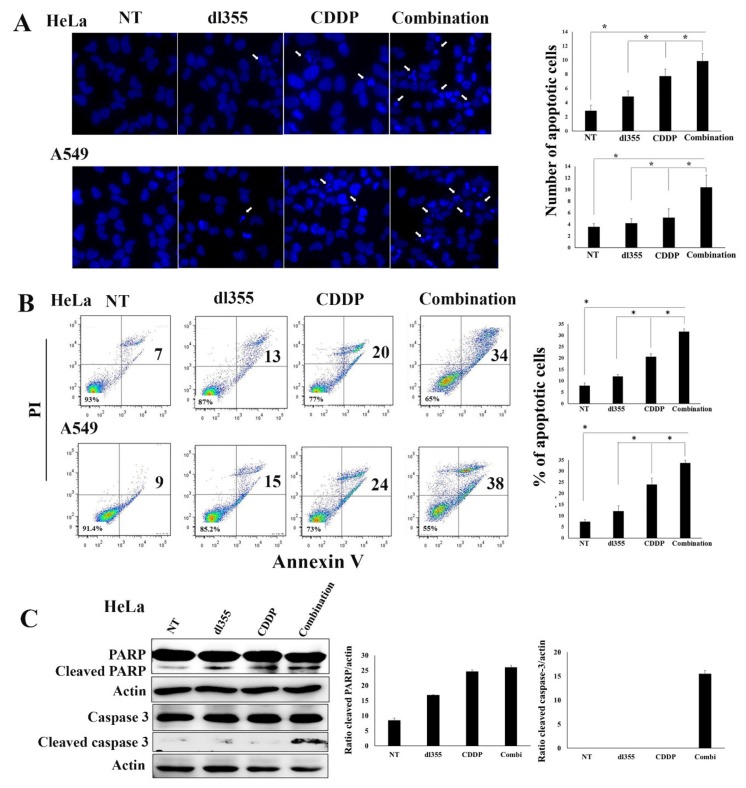
Evaluation of apoptosis induction of tumor cells by combination therapy. HeLa and A549 cells were treated with dl355 (MOI 10 vp/cell) or CDDP (1.25ug/mL) or combination (CDDP 1.25 µg/mL plus dl355 MOI 10; vp/cell) therapy for 72 h. For combination therapy, cells were treated with CDDP for 4 h before infection with the virus. The induction of the caspase pathway is observed only in the HeLa cell line. (**A**). Hoechst 33342 staining (1 mg/mL) for 30 minutes was used to detect apoptosis. Arrow marks indicate the condensed and fragmented nuclei. The number of apoptotic cells is quantitated in the histogram (right panel). (**B**). After incubation with annexin V and propidium iodide (PI), apoptotic cell detection was done by flow cytometry. The percentage values are shown at the right of each plot belong to the two quadrants on the right. The number of apoptotic cells is quantitated in the histogram (right panel). (**C**). HeLa cell was treated with similar treatment, as mentioned above. Activation of the caspase pathway was detected by western blot analysis. The bar chart shows the quantification of the cleaved PARP and cleaved caspase-3 level compared to actin in each condition (right panel). The result shown is from a single experiment representative of two similar experiments. * indicates *p* < 0.05.

To investigate the underlying cell killing mechanism, HeLa and A549 cells were treated with dl355 or CDDP or combination therapy, and the apoptotic effect was assessed after 72 h of treatment. Hoechst 33342 staining was performed to detect apoptotic cells, which were characterized by apoptotic bodies, chromatin condensation, and nuclear fragmentation. As shown in Figure 4A, combination therapy resulted in significantly increased apoptosis compared to dl355 or CDDP treatment alone. These observations were consistent with the expression of morphological changes after combination treatment. A large number of tumor cell death was observed with combination therapy than any of the treatment alone (Figure S3). 

Apoptosis was also assessed by annexin V and PI staining by flow cytometry (Figure 4B). We observed a higher proportion of apoptotic cells when treated with the combination therapy. In HeLa cells, dl355 and CDDP induced 12.5% and 20% of apoptosis, respectively, whereas the combined treatment resulted in 34% of apoptosis. In A549 cells, dl355 and CDDP induced 14% and 24% of apoptosis, respectively, whereas the combination therapy occurred in 38% of apoptosis. As combination therapy showed a similar enhancement in apoptotic cell death in both cell lines, next, we evaluated the caspase apoptotic pathway in the HeLa cell line by western blot analysis. A higher expression of cleaved PARP and caspase-3 in cells was observed in combination therapy than in either treatment alone (Figure 4C). These results suggest that the enhanced killing of cancer cells by combination treatment is due to higher apoptosis induction.

### 2.5. Enhanced In Vivo Antitumor Effect in Combination Therapy

The antitumor efficacy of dl355, in combination with CDDP, was evaluated in a subcutaneous cervical xenograft model based on HeLa S3 cell implantation. After the injection of tumor cells, the mice were observed for 50 days, and tumor volume was measured as described.

**Figure 5:**
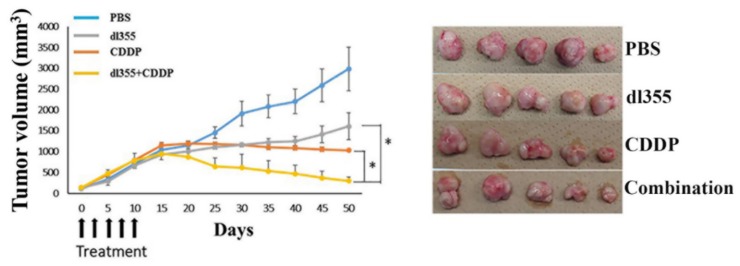
Evaluation of the therapeutic potential of combined therapy in vivo. The subcutaneous cervical xenograft model was developed based on HeLa S3 cell implantation. Treatments were as follows: PBS (control), dl355, CDDP, and combination. CDDP (intraperitoneal) and dl355 (intratumoral) injection was administered. We measured tumor volume at different time points after treatment. The image showed the differences in tumor volume on day 40 post-treatment when the mice were sacrificed. * indicates *p* < 0.05.

As shown in Figure 5, the mean tumor volume was decreased significantly in combination therapy compared to other treatment groups. By day 50, the average tumor volumes in each group were PBS (2984 mm^3^), dl355 (1609 mm^3^), CDDP (1032 mm^3^), and combination therapy (300 mm^3^). In addition, throughout the treatment, no systemic toxicity was observed in combination therapy.

### 2.6. CDDP - Induced In Vitro and In Vivo Virus Replication 

To confirm the effects of CDDP on viral replication, we performed an in vitro virus proliferation assay. HeLa and A549 cells were infected with dl355 and treated with combination therapy, as mentioned in the method section. Virus titers were analyzed 48 h after each treatment. Although a marked difference was not observed, the data showed increased dl355 replication in combination therapy than the dl355 alone (Figure S4).

**Figure 6:**
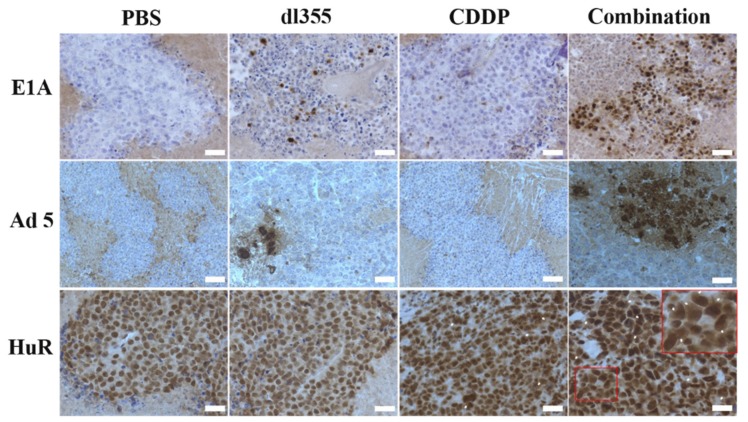
Effect of CDDP in virus replication and enhanced cytoplasmic HuR translocation. After fixation and paraffin processing, tumor specimens were sectioned at 4-μm thicknesses. The in vivo expression of E1A, Adenovirus type 5 capsid proteins, and HuR in treated and untreated tumors were examined by immunohistochemistry at magnification ×20. Adenovirus-infected cells were stained as brown. Arrow indicates cytoplasmic HuR expression. Inset shows (lower right panel) higher magnification of cytoplasmic HuR expression. Bars, 50 μm.

To assess the in vivo viral replication within the tumor, E1A and Adenovirus type 5 capsid proteins and HuR immunohistochemical staining were performed (animals treated with PBS or CDDP alone group were treated as negative control). An increase in viral protein expression was found in tumors of mice treated with combination therapy compared with those treated with dl355 alone. Besides, immunohistochemical staining of HuR protein demonstrated enhanced cytoplasmic HuR expression following CDDP and combination treatment in vivo. This finding indicates the effect of CDDP in cytoplasmic HuR translocation that enhance virus replication (Figure 6). 

## 3. Discussion

Enhanced antitumor activity was reported when adenoviruses are combined with chemotherapy or radiotherapy. Several reports suggested that chemotherapy or irradiation creates a favorable environment for enhanced viral replication [23,24,25]. Resistant to a single oncolytic agent or monotherapy is commonly reported as tumors are originated from diverse clones. Therefore, combinations of two or more therapeutic agents, each with a different antitumor mechanism, are highly recommended to achieve the synergistic antitumor efficacy [26,27]. 

CDDP is an alkylating drug that causes DNA damage by cross-linking with the purine bases on the DNA. This drug induces apoptosis in tumor cells by interfering with DNA repair mechanisms [28]. Recent reports have shown that oncolytic adenovirus and CDDP combination treatment results in increased cytotoxicity in human nasopharyngeal, lung, hepatocellular, ovarian, cervical, and colorectal cancer [29,30]. 

The molecular mechanism of intracellular HuR localization and constitutive cytoplasmic HuR accumulation of cancer cells is yet to be known. A previous study reported the cytoplasmic HuR translocation in response to stresses such as actinomycin D, hydrogen peroxide, or short-wavelength ultraviolet light without measurable influence on total or nuclear HuR levels. Data showed that nuclear HuR levels are almost 100% under normal circumstances, and only up to 20–30% of HuRs are exported to the cytoplasm under stress conditions. This negligible nuclear HuR reduction may not be detectable by western blot or confocal analysis [31]. We reported a similar finding where we showed microfilament dependent and independent cytoplasmic HuR translocation without changing the nuclear and total content [32]. Our findings agree with previous reports showing that HuR translocates to the cytoplasm in response to CDDP without attenuation of nuclear content (Figure 2B). In addition, we observed the granular SGs in CDDP treated cancer cells, which shows increased cytoplasmic HuR protein (Figure 2C). As stresses were reported to induce HuR accumulation into SGs, we hypothesized that the upregulation of cytoplasmic HuR by CDDP treatment might be linked to SG formation.

HuR stabilizes many target mRNAs, including those that encode stress-response and proliferative proteins [12,13,14,15,31] Reports show the evidence that stresses induced post-transcriptional effect of HuR on its target mRNAs that are correlated to cytoplasmic HuR translocation [33]. In a previous study, we reported that HuR plays an essential role in adenovirus replication via the stabilization of the IVa2 mRNA. Besides, levels of the IVa2 mRNA also decreased in HuR-depleted cells by heat shock and knocking down HuR [11]. Similar findings were also reported the reduced dl355 replication in HuR depleted cells [22]. As the role of HuR in ARE mRNA stabilization is already published, we assumed that CDDP induced HuR exportation increased various ARE mRNA as reported previously. However, CDDP influenced increased ARE mRNA should be evaluated in the future study. 

From the above reports, we assumed that in response to CDDP induced stress, HuR translocates to the cytoplasm leads to ARE mRNA stabilization required for enhanced dl355 replication. Consistent with those mechanisms, our in vitro data demonstrated that, compared with the single therapy, combination treatment led to a marked oncolytic effect (Figure 3 and Figure S2). Besides, Hoechst 33342 staining, flow cytometry, and western blot data reveal that in combination therapy, cell death is mainly occurred by apoptosis due to the activation of the intrinsic apoptotic pathway (Figure 4). To evaluate the effect of CDDP on viral replication, cancer cells (HeLa and A549) were infected with dl355 and treated with combination therapy. Increased virus titer in combination therapy indicates enhanced virus replication (Figure S4).

Moreover, the superior oncolytic impact and increased virus propagation of combination therapy were demonstrated in an in vivo model (Figure 5 and Figure 6). Immunostaining of E1A, capsid proteins and HuR was performed to assess the virus extension and cytoplasmic HuR translocation in in vivo tumor samples. Enhanced expression of E1A and capsid protein in combination therapy indicates the presence and replication of the virus, respectively, even after 40 days of treatment completion. Besides, enhanced cytoplasmic HuR expression following CDDP and combination treatment reconfirms the effect of CDDP in cytoplasmic HuR translocation (Figure 6). 

Although oncolytic virotherapy kills cancer cells more specifically, it has not been effective so far as a single therapy due to poor dissemination and limited ability to transduction within tumors [34]. At present, several clinical trials reported significantly enhanced antitumor activity when incorporating irradiation or chemotherapy to virotherapy [35,36,37,38,39]. Therefore, a detailed study is required to understand the interactions between viruses and chemotherapeutic agents to design more effective anticancer drugs [40].

## 4. Materials and Methods 

### 4.1. Cell Lines, Culture Conditions, and Reagents 

The human cervical carcinoma cells HeLa, HeLa S3 and C33A, lung carcinoma cells A549 and H1299, osteosarcoma cells U2OS, tongue carcinoma cell HSC3, gingiva carcinoma cell Ca922, normal foreskin fibroblast cell BJ, normal lung primary cell WI38, and African green monkey kidney (Vero) cells carrying an integrated copy of the Ad5 E4 region, W162 were used. Cells were purchased from American Type Culture Collection (ATCC; Manassas, VA, USA) and cultured in Dulbecco’s modified Eagle’s medium (DMEM; Sigma-Aldrich, Germany) containing 10% fetal bovine serum (FBS, France) with antibiotics at 37 °C in a 5% CO_2_ atmosphere humidified conditions.

CDDP was obtained from Pfizer Pharmaceutical Group (New York, NY, USA). Cell proliferation kit II was purchased from Roche Diagnostics (Germany). FITC Annexin V and Propidium Iodide Solution (PI) were purchased from Biolegend (San Diego, CA, USA). 

### 4.2. Preparation of Virus Lysates

E4orf6-deleted mutant adenovirus (dl355) was used in this study. To prepare virus lysates, dl355-infected W162 cells were subjected to three cycles of freezing and thawing. Virus concentrations (virus particles; vp/mL) and titers (infectious units (ifu)/mL) were determined by the Adenovirus Quantitation kit (Cell Biolabs, CA, USA) and Adeno-X™ Rapid Titer kit (Clontech Laboratories, Inc., CA, USA) respectively. For in vivo experiments, the viral extract was purified by a Fast-Trap Adenovirus Purification and Concentration Kit (Millipore, Billerica, MA, USA).

### 4.3. Western Blot Analysis

The total cell lysates were prepared using radioimmunoprecipitation assay (RIPA) buffer. To separate cells into cytoplasmic and nuclear fractions, fractionating buffer was used. Proteins were separated by 10% sodium dodecyl sulfate-polyacrylamide gel electrophoresis (SDS-PAGE) and transferred onto polyvinylidene difluoride membranes (PVDF) (Millipore, Billerica, MA, USA). Specific proteins were analyzed by incubating the membrane with primary antibodies as follows: HuR (dilution 1:2,500, cat no sc-5261, Santa Cruz Biotechnology, Oregon, USA), β-tubulin (dilution 1:1,000; cat. no. 05-661; EMD Millipore Corp., Darmstadt, Germany), actin (dilution 1:1,000; cat. no. sc-1616; Santa Cruz Biotechnology, Dallas, TX, USA), Lamin B (dilution 1:1,000; cat no sc-6216, Santa Cruz Biotechnology, Dallas, TX, USA), PARP (dilution 1:1000; cat. no. 9532, Danvers, MA, USA), caspase-3 (dilution 1:1000; cat. no. 9662; Cell Signaling Technology, Danvers, MA, USA). The following secondary antibodies were used: horseradish peroxidase-conjugated anti-goat IgG (dilution 1:5,000; cat. no. 805-035-180; Jackson Immuno Research Laboratories, PA, USA) and horseradish peroxidase-conjugated anti-mouse IgG (dilution 1:5,000; cat. no. 115-035-062; Jackson Immuno Research Laboratories, PA, USA). The bands were visualized using the Supersignal West Femto Maximum Sensitivity Substrate (Thermo Fisher Scientific, Inc., Waltham, MA, USA). 

### 4.4. Fluorescence Microscopy

HeLa and A549 cells were treated with CDDP (1.25 µg/mL) at the indicated time (4, 8, 12, and 16 h). After fixation, blocking and permeabilization, the cells were incubated with the anti-HuR primary antibody, followed by Alexa Fluor 488 secondary antibodies. Cell nuclei were stained with DAPI before mounted on slides by using Mountant permafluor (Thermo scientific, FM 111212A). Cells were observed using an IX71 inverted microscope, and image acquisition was performed with the Olympus Fluoview Software (FV10-ASW 4.2 viewer).

### 4.5. Cytopathic Effect Assay

Cancer and normal cells were seeded in 24-well dishes with a density of 5 × 10^4^ cells/well 24 h and then infected with dl355 with varying concentrations of the multiplicity of infection (MOI) (MOI; 0.1, 0.5, 1, or 10; vp/cell) and incubated for seven days. To evaluate the efficacy of combination therapy, cells were treated with CDDP (1.25 µg/mL) or dl355 (MOI 10; vp/cell) or combination therapy (CDDP 1.25µg/mL plus dl355 M 10; vp/cell) and incubated for 5 days. For combination therapy of H1299, MOI 1 was used instead of MOI 10 (since all cells died at MOI 10 on day 7). After the incubation period, plates were washed with phosphate-buffered saline (PBS), fixed and stained with coomassie brilliant blue.

### 4.6. XTT Assay and Chou-Talalay Analysis

Viability of dl355 and CDDP were first evaluated by XTT assay (A 2-3-bis [2-methoxy-4-nitro- 5-sulfophenyl] -2H-tetrazolium-5-carboxanilide inner salt assay). Briefly, HeLa and A549 cells were seeded on 96-wells with a density of 3.0 × 10^3^ cells/well. The wells were incubated with varying concentrations dl355 (M 1-1000; vp/cell) or CDDP (.3 to 20 µg/mL) for 48 h. Cell viability was determined by XTT assay. For combination therapy, cells were seeded and treated with CDDP (1.25 µg/mL). At 4 h post-treatment with CDDP, cells were infected with dl355 (MOI 10; vp/cell), and cell viability was assessed on days 2, 4, and 6 of incubation. To evaluate the interactions between CDDP and dl355, HeLa and A549 cells were treated with the varying drug (CDDP; 0.6, 1.25 and 2.5 µg/mL) and viral (dl355; MOI 5, 10, and 50 vp/cell) concentrations for 5 days. Chou-Talalay combination indices (CI) were calculated using CompuSyn software (ComboSyn). 

### 4.7. Hoechst 33342 Staining

HeLa and A549 cells were treated with dl355 (MOI 10; vp/cell) or CDDP (1.25 µg/mL) or combination therapy (CDDP 1.25 ug/mL plus dl355 M 10; vp/mL) and incubated for 72 h. Cells were further incubated with Hochest 33342 for 30 minutes. Apoptotic cells were observed under a fluorescence microscope. 

### 4.8. Flow Cytometry Analysis 

HeLa and A549 cells were treated with dl355 (MOI 10; vp/cell) or CDDP (1.25 µg/mL) or combination therapy (CDDP 1.25 µg/mL plus dl355 M 10; vp/cell) and incubated for 72 h. Apoptosis was indicated by fluorescein isothiocyanate (FITC)-labeled annexin V and PI staining (BioVision, Palo Alto, CA, USA) as per manufacturer’s instructions. The stained cells were analyzed using FACS (BD FACS Canto TM II Flow cytometer, BD Biosciences, CA, USA) and FlowJo version 7.6 (Tomy Digital Biology Co., Ltd, Japan).

### 4.9. In Vitro Virus Proliferation Assay

HeLa and A549 cells were treated with dl355 (MOI 10; vp/cell) or combination therapy (CDDP 1.25 µg/mL plus dl355 M 10; vp/cell) and incubated for 48 h at 37 °C. After the incubation period, cells were collected, and a virus lysate was prepared as described above. Adeno-X Rapid Titer kit (Clontech Laboratories) was used to determine the viral titers (ifu/mL).

### 4.10. In Vivo Antitumor Effect

For in vivo experiments, female BALB/c nu/nu mice (purchased from Hokudo, Sapporo, Japan) were housed under specific pathogen-free conditions. The temperature (26–28 °C) and the light/dark cycle was maintained.

HeLa S3 cells (1.0 × 10^6^ cells/mouse) were inoculated subcutaneously into the right flanks of mice (5 weeks). When tumors measured an average volume of 100–150 mm^3^, the mice were divided into four groups a, b, c, d (five-mice in each group). Group a received PBS (days, 22, 24, 26, 28 and 29), group b was treated with dl355 (days, 23, 25, 27, 29 and 31; 10^8^ vp [100 μL] intratumorally), group c was treated with CDDP (days 22, 24, 26, 28 and 29; 2 mg/kg; intraperitoneally) and group d was treated with combination therapy (CDDP on days 22, 24, 26, 28 and 29 followed by dl355 on days 23, 25, 27, 29 and 31). Tumor growth was measured every 3 days using Vernier calipers. Tumor volume was estimated based on the following formula: Volume (mm^3^) = A × B2 × 0.5 (A is length, B is the width of the tumor). The mice were sacrificed by cervical dislocation. All procedures performed in this study were under the ethical standards committee of Animal Care. Hokkaido University. Sapporo, Japan (Permission number for the animal experiment: 13-0058).

### 4.11. Immunohistochemistry Analysis 

The xenografted tumors were removed, fixed with 4% formaldehyde, and embedded for hematoxylin and eosin staining and immunohistochemistry. Next paraffin-embedded specimens were sectioned in four-μm-thick. Detection of the virus was done using mouse anti-E1A (M73; 1:1000) and rabbit anti-capsid (1:2000; Abcam, Cambridge, UK) antibodies. HuR expression was detected with mouse anti-HuR antibodies (1:5000 dilution; Santa Cruz Biotechnology, Oregon, USA). The slides were incubated with diaminobenzidine (DAB) chromogen and counterstained with hematoxylin for microscopic observation. 

### 4.12. Statistical Analysis

Statistical analysis was performed using one-way ANOVA. Post-hoc multiple comparisons were done by Tukey’s test at a 5% level of significance. * indicates *p* < 0.05.

## 5. Conclusions

This study demonstrated that CDDP induces the HuR accumulation into stress granule, and the upregulation of cytoplasmic HuR is linked to mRNA stabilization and enhanced dl355 replication. Our results showed that dl355 could effectively infect and kill various cancer cell lines. Besides, combination therapy increases apoptosis and cell death in tumor cells without increased lethality in normal cells. Therefore, the use of CDDP and dl355 as a combination could be an attractive strategy to improve the therapeutic potential of cancer treatment.

## Supplementary Materials

The supplementary figures are available online at https://www.mdpi.com/2072-6694/12/4/809/s1, Figure S1: Cytotoxicity of combination therapy and Chou-Talalay analysis, Figure S2: Cell viability assessment after dl355 infected and combination therapy, Figure S3: Expression of morphological changes after dl355 infection and CDDP treatment. Figure S4: Effect of CDDP in virus replication in combination therapy. The uncropped blots and molecular weight markers are shown in Supplementary Materials.
