# Supplementary material for: Cisplatin Relocalizes RNA Binding Protein HuR and Enhances the Oncolytic Activity of E4orf6 Deleted Adenovirus
Source: Cancers (Basel). 2020 Mar 27;12(4):809. doi: 10.3390/cancers12040809 (PMC7226092; doi:10.3390/cancers12040809)
Supplement: Supplementary file 1 [file cancers-12-00809-s001.zip › cancers-724145.supp.docx]

**
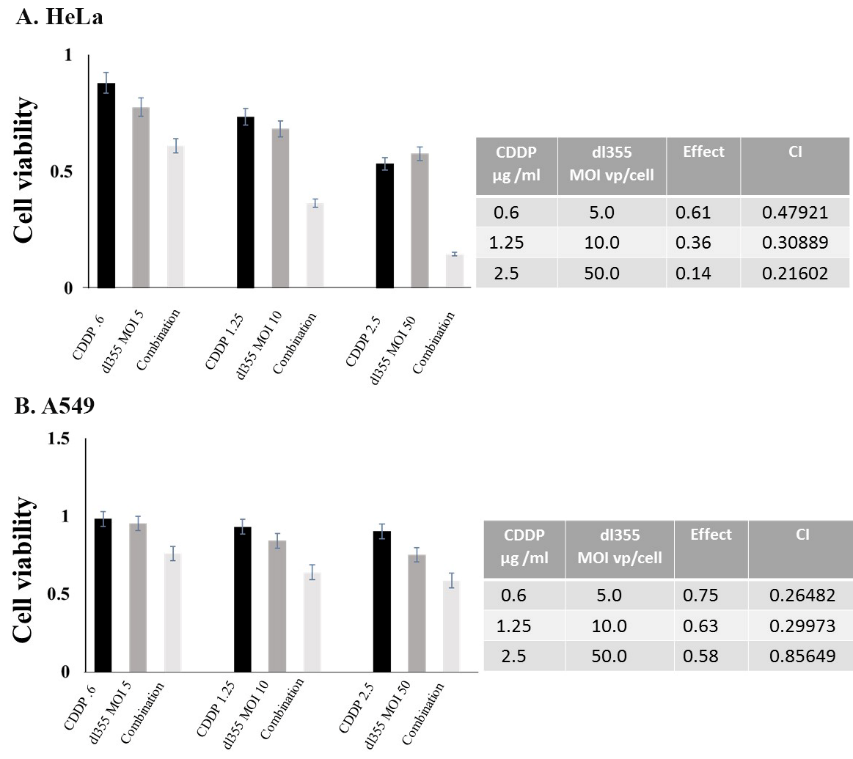
**

**Figure S1.** Cytotoxicity of combination therapy and Chou-Talalay analysis.

Cytotoxic effect of combination therapy on HeLa and A549 cells using XTT assay.Varying drug (CDDP; .6,1.25 and 2.5 µg /mL) and viral (dl355; MOI 5,10, and 50 vp/cell) concentrations were used alone or in combination for 5 days. The CIs of the drug, in combination with the virus, were calculated using Chou-Talalay analysis.CI of <0.9 indicates synergy, CI between 0.9 and 1.1 is addictive, and CI of >1.1 indicates antagonism.


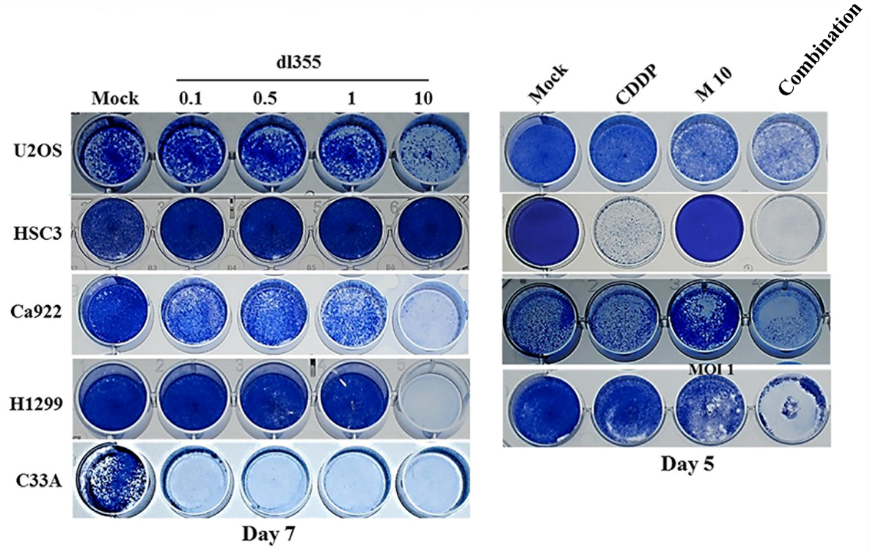


**Figure S2.** Cell viability assessment after dl355 infected and combination therapy. Cancer cells were infected with dl355 at indicated MOIs (0.1, 0.5, 1, 10; vp/cell) or treated with CDDP (1.25 µg /mL) or combination (CDDP 1.25 µg /mL plus dl355 MOI 10; vp/cell) therapy. For combination therapy, cells were treated with CDDP for 4 h before infection with the virus. Since all cells died at MOI 10, on day 7, for combination therapy of H1299, MOI 1 was used instead of MOI 10. At 5 and 7 days of post-treatment, coomassie brilliant blue staining was used to evaluate the cytopathic effect. The result shown is from a single experiment representative of two similar experiments.* indicates p < 0.05.

**
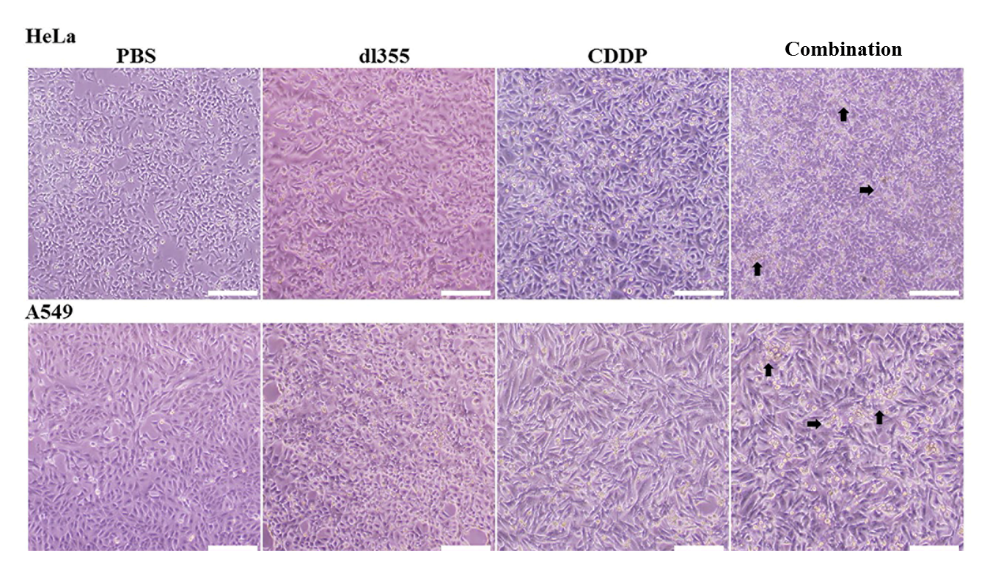
**

**Figure S3.** Expression of morphological changes after dl355 infection and CDDP treatment. HeLa and A549 cells were treated with dl355 (MOI 10 vp/cell) or CDDP (1.25ug/mL) or combination (CDDP 1.25 µg /mL plus dl355 MOI 10; vp/cell) therapy for 72 h. For combination therapy, cells were treated with CDDP for 4 h before infection with the virus. The morphological change was seen under the microscope in each treatment, and marked cell death was observed in combination therapy (marked with an arrow) than any of the treatment alone.

**
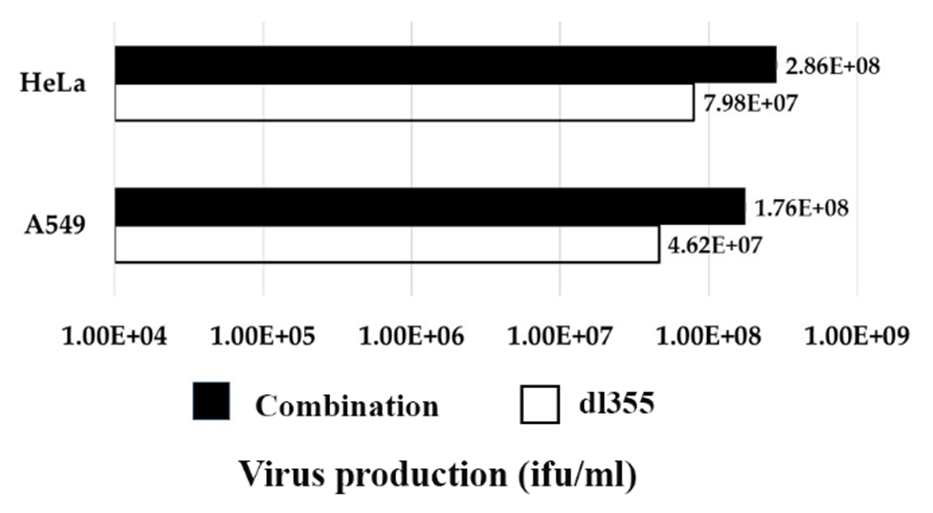
**

**Figure S4.** Effect of CDDP in virus replication in combination therapy. HeLa and A549 cells were treated with dl355 (MOI 10 vp/cell) or combination (CDDP 1.25 µg /mL plus dl355 MOI 10; vp/cell) therapy and virus production was determined 48 h after infection by hexon staining. Each titer (ifu/mL) is indicated on the graph. Data are shown as the mean ± standard deviation of two independent experiments.
