# Supplementary figures and images for: Cisplatin Relocalizes RNA Binding Protein HuR and Enhances the Oncolytic Activity of E4orf6 Deleted Adenovirus
Source: Cancers (Basel). 2020 Mar 27;12(4):809. doi: 10.3390/cancers12040809 (PMC7226092; doi:10.3390/cancers12040809)

**Figure 2B ; whole blot with marker**

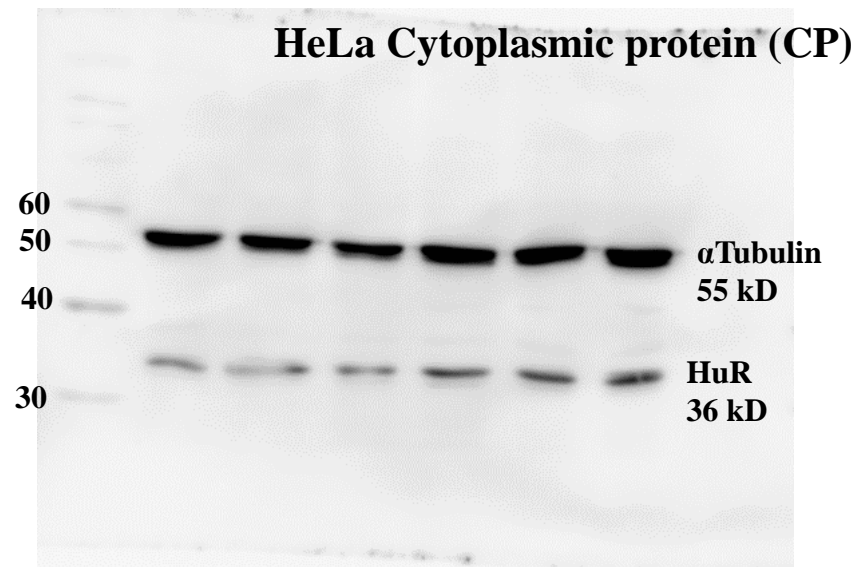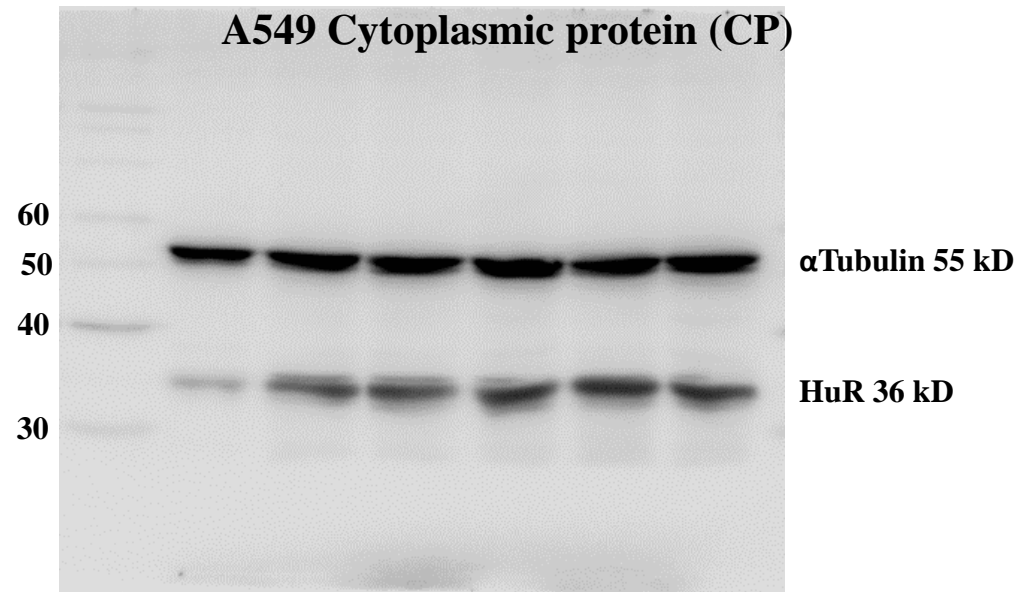

**Figure 4C; whole blot with marker**

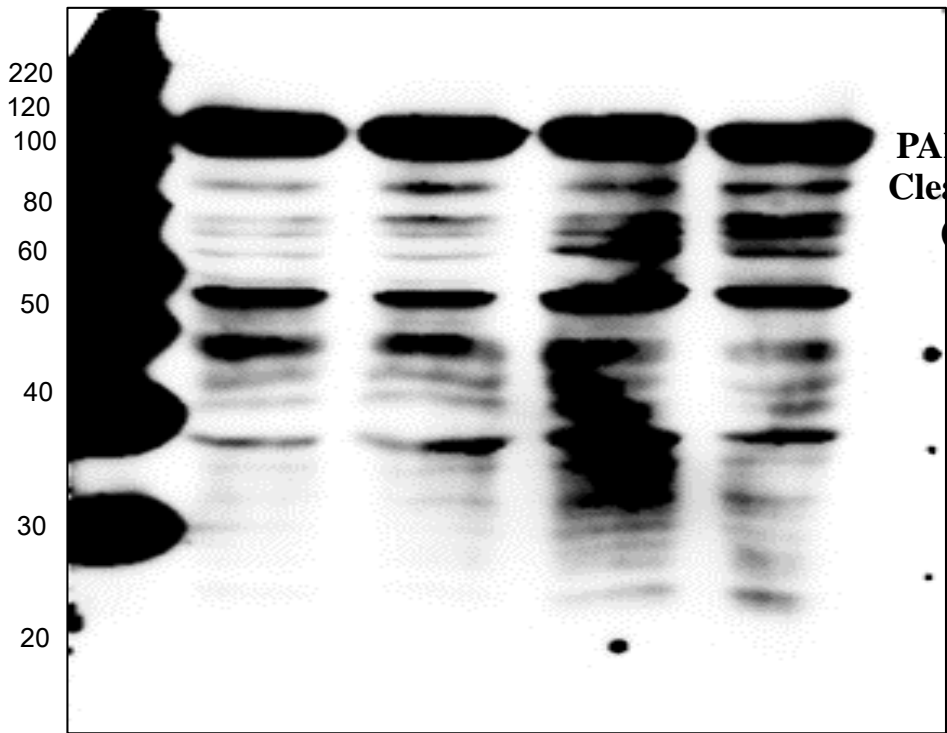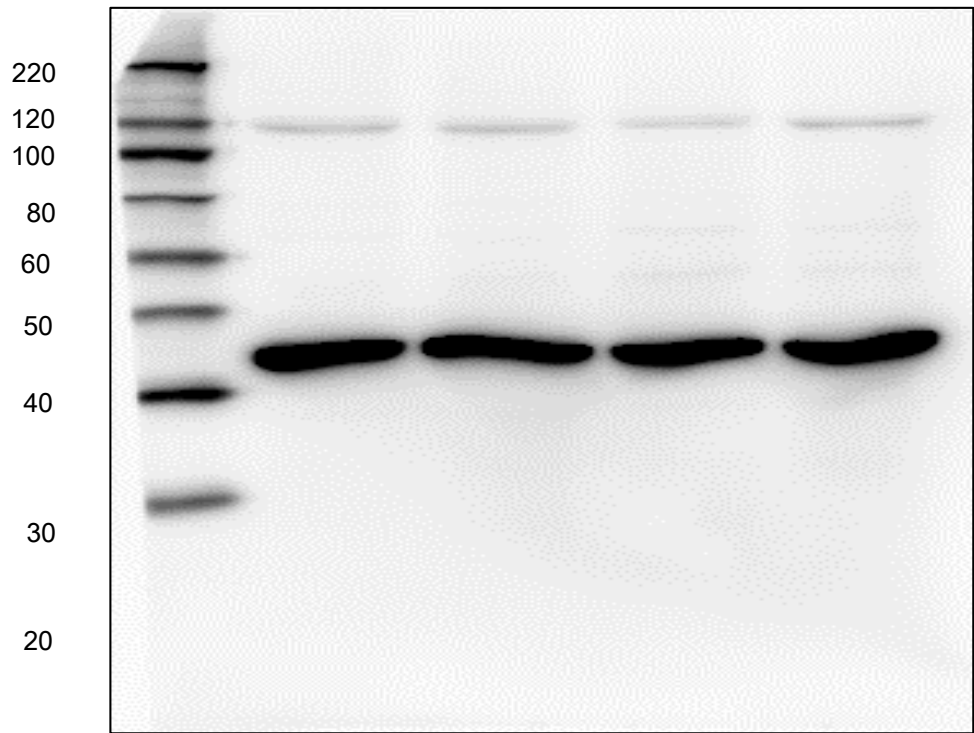

**Figure 4C; whole blot with marker**

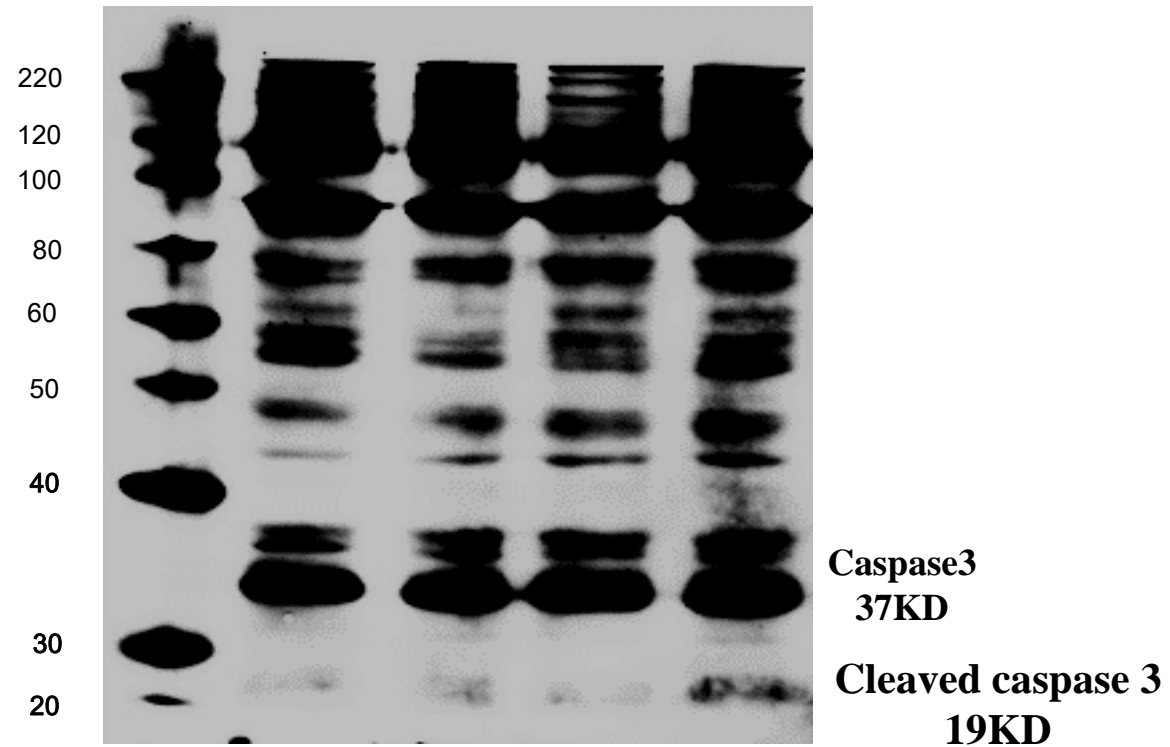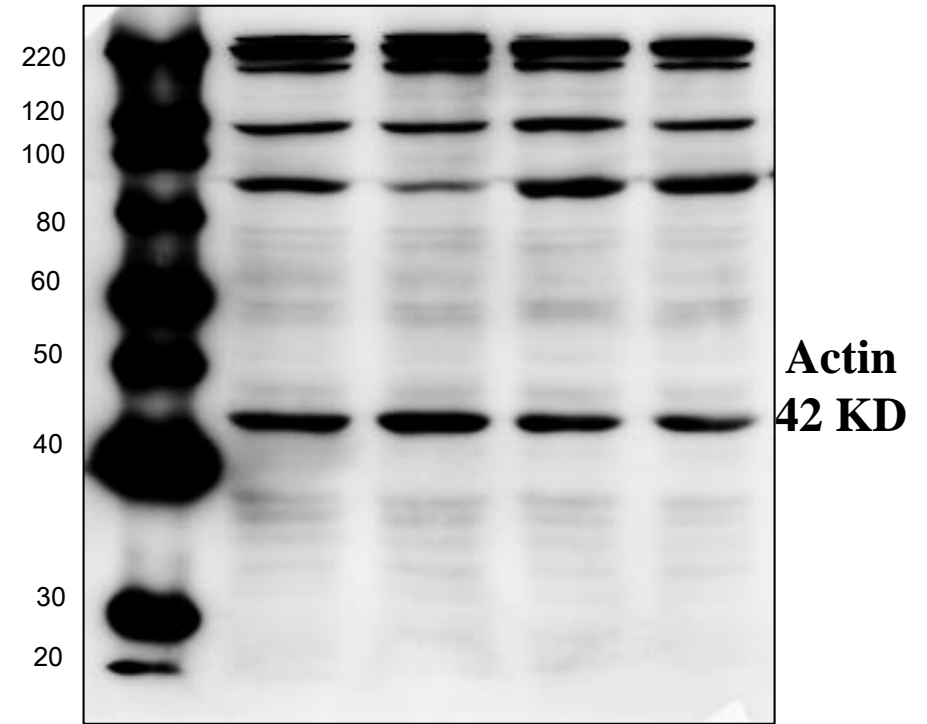

Supplement: Supplementary file 1 [file cancers-12-00809-s001.zip › cancers-724145.non-published materials.pdf]
